# Editorial Note: Complete Phenotypic Recovery of an Alzheimer’s Disease Model by a Quinone-Tryptophan Hybrid Aggregation Inhibitor

**DOI:** 10.1371/journal.pone.0335097

**Published:** 2025-10-23

**Authors:** The PLOS One Editors

After this article [1] was published, concerns were raised about Figs 1 and 2. Specifically:

- There appears to be a vertical discontinuity between lanes 1 and 2 in Fig 1B.
- Fig 2C appears similar to Fig 1D in a later article [2].

In response, corresponding author DS stated that the data in Fig 1B were generated from one gel in which multiple inhibitors (both quinone inhibitors) were tested; the control was run on the same gel and was used for both sets of inhibitor conditions; and for clarity in presentation, the control lane was cropped and placed adjacent to the corresponding inhibitor concentrations.

Regarding Fig 2C, corresponding author DS stated that Fig 2C in [1] and Fig 1D in [2] are intentionally the same, as both panels are control microscope images representing the same THT experiment in which two different inhibitors were tested. They stated that the control image in Fig 2C in [1] was used as a reference for both inhibitors as all analyses were conducted within a single, comprehensive experiment, and that the reuse of the control image as Fig 1D in [2] accurately reflects the experimental design and data. Given the information provided, the *PLOS One* Editors consider the above concerns resolved.

Corresponding author DS also stated that the original data underlying [1] are no longer available.

The *PLOS One* Editors issue this Editorial Note to give readers the above information about Figs 1 and 2 in [1].
